# EPDR1 is a noncanonical effector of insulin-mediated angiogenesis regulated by an endothelial-specific TGF-β receptor complex

**DOI:** 10.1016/j.jbc.2022.102297

**Published:** 2022-07-21

**Authors:** Tasmia Ahmed, Paola Cruz Flores, Christopher C. Pan, Hannah R. Ortiz, Yeon S. Lee, Paul R. Langlais, Karthikeyan Mythreye, Nam Y. Lee

**Affiliations:** 1 Department of Chemistry & Biochemistry, University of Arizona, Tucson, Arizona, USA; 2 Department of Pharmacology and Cancer Biology, Duke University, Durham, North Carolina, USA; 3 Department of Pharmacology, University of Arizona, Tucson, Arizona, USA; 4 Department of Medicine, University of Arizona, Tucson, Arizona, USA; 5 Department of Pathology, University of Alabama at Birmingham, Birmingham, Alabama, USA; 6 Comprehensive Cancer Center, University of Arizona, Tucson, Arizona, USA

**Keywords:** TGF beta, angiogenesis, insulin, Smad, endothelial biology, EC, endothelial cell, FBS, fetal bovine serum, HA, hemagglutinin, IF, immunofluorescence, IP, immunoprecipitation, IR, insulin receptor, IRS, insulin receptor substrate, MEEC, mouse embryonic EC, MS, mass spectrometry, TBS-T, Tris-buffered saline with Tween-20

## Abstract

Insulin signaling in blood vessels primarily functions to stimulate angiogenesis and maintain vascular homeostasis through the canonical PI3K and MAPK signaling pathways. However, angiogenesis is a complex process coordinated by multiple other signaling events. Here, we report a distinct crosstalk between the insulin receptor and endoglin/activin receptor-like kinase 1 (ALK1), an endothelial cell–specific TGF-β receptor complex essential for angiogenesis. While the endoglin–ALK1 complex normally binds to TGF-β or bone morphogenetic protein 9 (BMP9) to promote gene regulation *via* transcription factors Smad1/5, we show that insulin drives insulin receptor oligomerization with endoglin–ALK1 at the cell surface to trigger rapid Smad1/5 activation. Through quantitative proteomic analysis, we identify ependymin-related protein 1 (EPDR1) as a major Smad1/5 gene target induced by insulin but not by TGF-β or BMP9. We found endothelial EPDR1 expression is minimal at the basal state but is markedly enhanced upon prolonged insulin treatment to promote cell migration and formation of capillary tubules. Conversely, we demonstrate EPDR1 depletion strongly abrogates these angiogenic effects, indicating that EPDR1 is a crucial mediator of insulin-induced angiogenesis. Taken together, these results suggest important therapeutic implications for EPDR1 and the TGF-β pathways in pathologic angiogenesis during hyperinsulinemia and insulin resistance.

Transforming growth factor β (TGF-β) is a multifunctional cytokine that affects nearly every aspect of mammalian physiology starting from embryogenesis and tissue differentiation to adult homeostasis (1, 2). It plays an essential role in angiogenesis during embryonic development mediated in large part by endoglin, an endothelial cell (EC)–specific coreceptor that binds TGF-β and presents it to the signaling kinase receptor, ALK1, which in turn induces proangiogenic effects through Smad1/5-dependent transcriptional responses (3, 4, 5, 6). In the absence of endoglin expression, TGF-β signals mainly through ALK5, a ubiquitously expressed receptor kinase that promotes Smad2/3-dependent cell growth arrest and vascular quiescence (7, 8). Given these specialized angiogenic functions, endoglin and ALK1 are viewed as particularly effective vascular targets to complement the inhibition of vascular endothelial growth factor receptor (VEGFR) signaling in a broad range of clinical applications including tumor growth, diabetic retinopathy, and age-related macular degeneration (5, 9, 10, 11).

Insulin is a primary regulator of glucose metabolism but also has a central role in angiogenesis and hemodynamics (12, 13). In ECs, insulin binding to the insulin receptor (IR) induces the downstream PI3K/Akt and MAPK pathways to enhance survival mechanisms while promoting nitric oxide (NO) production, cell proliferation, and vascular permeability (12, 13, 14, 15, 16, 17, 18). These proangiogenic effects have been demonstrated in various *in vitro* and *in vivo* models, although the underlying molecular mechanisms have yet to be fully established. There are several outstanding questions regarding IR signaling in the endothelium including how it spatiotemporally coordinates the VEGF-dependent and VEGF-independent mechanisms during sprouting angiogenesis, and perhaps more important, why IR signaling is critically important when in fact its canonical PI3K/Akt and MAPK pathways can be theoretically compensated by many other receptor tyrosine kinases including VEGFR2 and insulin-like growth factor 1 receptor (IGF-1R) (5, 10, 19, 20).

In defining new roles of insulin/IR signaling in the vasculature, here we report a direct crosstalk between the IR and the endoglin–ALK1 complex critical for angiogenesis. We find that insulin can signal though endoglin–Alk1 in ECs to trigger Smad1/5 transcriptional activation. Through quantitative proteomics, we identify ependymin-related protein 1 (EPDR1) as a powerful downstream gene target uniquely responsive to insulin but not TGF-β or BMP9. Moreover, we present molecular and cellular evidence of the underlying crosstalk mechanisms and the previously unknown role of EPDR1 in endothelial functions.

## Results

### Insulin induces rapid Smad1/5 activation in vascular ECs

**Figure 1:**
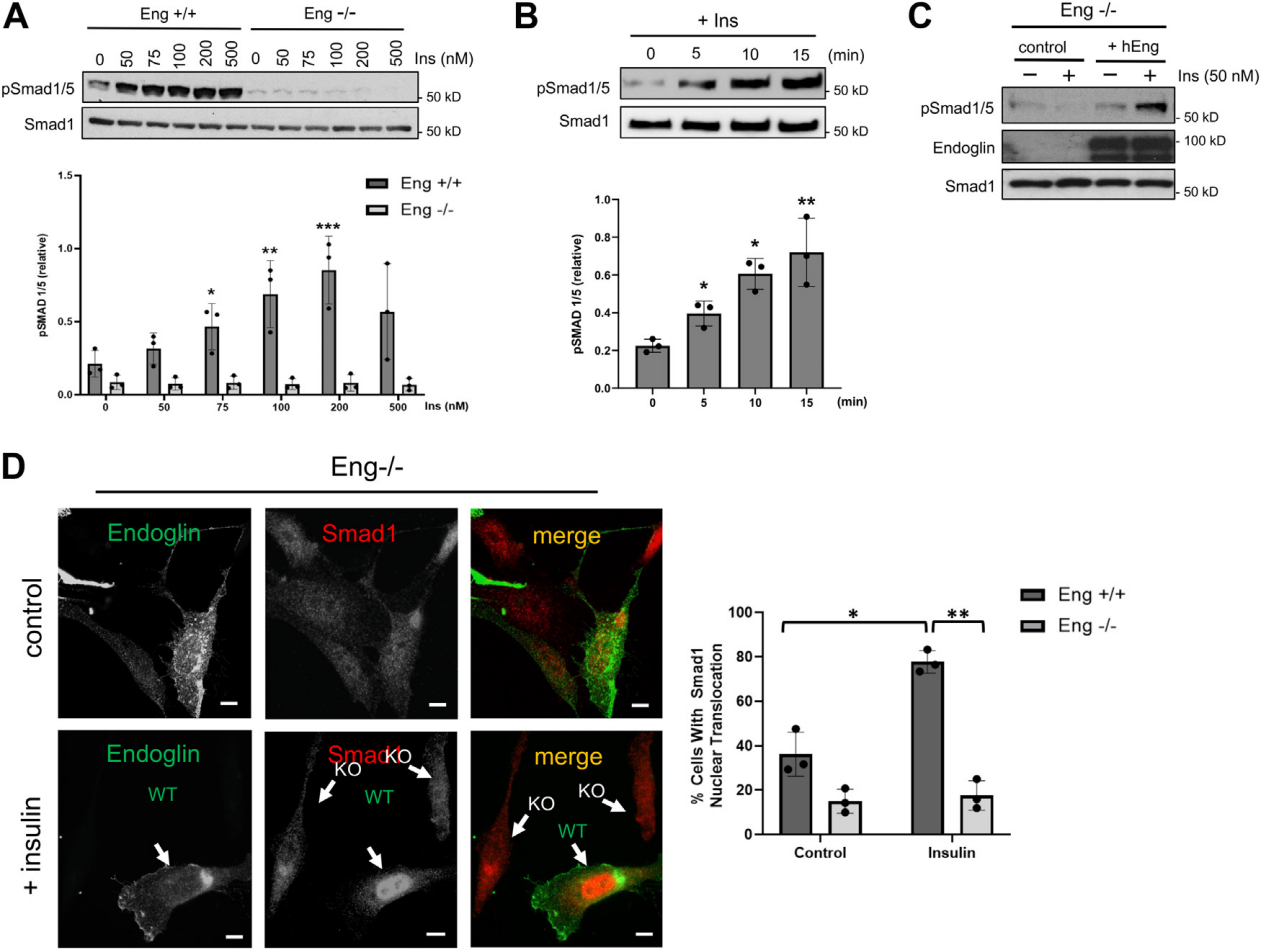
**Insulin-induced Smad1/5 activation requires endoglin expression in ECs.***A*, Western blot shows pSmad1/5 levels in response to insulin at indicated concentrations in Eng+/+ and Eng−/− MEECs for 30 min. Cells were serum starved for 4 to 6 h prior to stimulation. Data are representative of three independent experiments. Blots were analyzed by densitometry and pSmad1/5 ratio to total Smad1 was quantified at different insulin concentrations. Error bars indicate mean with SD and type 2 *t* test result shows ∗*p* < 0.05, ∗∗*p* < 0.03, ∗∗∗*p* < 0.02 relative to Eng +/+ control (no treatment). *B*, Western blot shows pSmad1/5 levels at indicated time points upon insulin (100 nM) treatment in Eng+/+ MEECs with prior 6 h serum starvation. Data are representative of three independent experiments. Densitometry ratio of pSmad1/5 to total Smad1 was quantified at different insulin concentrations. Error bars indicate mean with SD and type 2 *t* test result shows ∗*p* < 0.05, ∗∗*p* < 0.03 relative to control (no treatment). *C*, Western blot shows pSmad1/5 levels upon insulin stimulation for 30 min in Eng−/− cells with or without ectopic overexpression of human endoglin (hEng) or vector control. *D*, representative immunofluorescence images show Eng−/− cells transiently transfected with endoglin expression vector. Serum starved cells (4–6 h) were treated with PBS control or insulin (30 min) before fixing, then stained for endoglin (*green*) and endogenous Smad1 (*red*). *White arrows* indicate endoglin-positive (WT) or endoglin KO cells and their Smad1 distribution. Graph represents percentage of endoglin-positive cells showing strong Smad1 nuclear translocation. About 25 cells were analyzed per group from three independent experiments. The scale bar represents 10 μm. Error bars indicate mean with SD and type 2 *t* test result shows ∗*p* < 0.05, ∗∗*p* < 0.005 relative to Eng+/+ control (no treatment). EC, endothelial cell; MEEC, mouse embryonic EC.

Previous studies have established important links between insulin and TGF-β signaling, most notably recent findings showing that insulin increases TGF-β responsiveness by promoting the cell surface retention of the TGF-β receptors (ALK5 and TβRII) (21, 22). While such effects were Akt dependent and caused Smad2/3 activation in diverse cell types including ECs (23), we sought an alternative mechanism specifically involving endoglin that could potentially explain why insulin causes Smad1/5 activation in vascular ECs. Indeed, we unexpectedly observed that insulin triggers Smad1/5 activation in both a dose-dependent and endoglin-dependent manner as evidenced by the increased Smad1/5 phosphorylation in control (Eng+/+) but not endoglin KO (Eng−/−) mouse embryonic ECs (MEECs) (Fig. 1*A*; graph). This induction was rapid with a discernable increase in phosphorylation occurring within 5 min of stimulation and reaching maximum levels upon 15 to 30 min before gradually decreasing over the course of 2 to 4 h (Fig. 1*B*; graph, Fig. S1*A*). To test whether endoglin directly mediates this response, we ectopically restored endoglin expression in Eng−/− ECs to find a distinct endoglin-dependent Smad1/5 phosphorylation upon insulin treatment, suggesting that endoglin is required for insulin-induced Smad1/5 phosphorylation (Fig. 1*C*). Finally, to test whether this effect is observed in other EC types, we chose a human microvascular EC (HMEC1) to assess whether Smad1/5 activation occurs in response to insulin in an endoglin-dependent manner. Similar to the MEECs, there was a rapid increase in Smad1/5 phosphorylation in control cells but not upon stable knockdown of endoglin expression (sh-Eng) (Fig. S1, *B* and *C*), thus demonstrating that endoglin mediates insulin-induced Smad1/5 phosphorylation in vascular ECs.

Because Smad1/5 phosphorylation causes their nuclear translocation for gene regulation (24), we next evaluated the effects of insulin on Smad1 subcellular localization. To do so, endoglin expression was ectopically restored in Eng−/− cells and then stimulated with insulin for 30 min prior to fixation for immunofluorescence (IF) analysis. At basal state, less than 10% of the Eng−/− cell population displayed Smad1 nuclear accumulation compared to roughly 30% in those transiently expressing endoglin (Fig. 1*D*; graph). However, insulin treatment caused markedly increased Smad1 translocation to nearly 80% in endoglin-positive cell populations compared to no change observed in endoglin-deficient ECs (Fig. 1*D*; graph). Together, these results indicated that the insulin-induced Smad1/5 activation and nuclear translocation strictly requires endoglin expression.

### Insulin promotes endoglin/IR interaction at the cell surface

Since insulin is capable of binding IR and IGF-1R (12, 18), we first tested whether IR is the primary mediator of Smad1/5 activation by generating a stable IR knockdown in MEECs (sh-IR; Fig. S2*A*). Subjecting these cells to insulin treatment showed impaired Smad1/5 phosphorylation over a broad range of insulin concentrations relative to control (Fig. S2*B*), thus indicating that insulin signals through IR. Based on this finding, we explored the possibility of a physical interaction between endoglin and IR.

**Figure 2:**
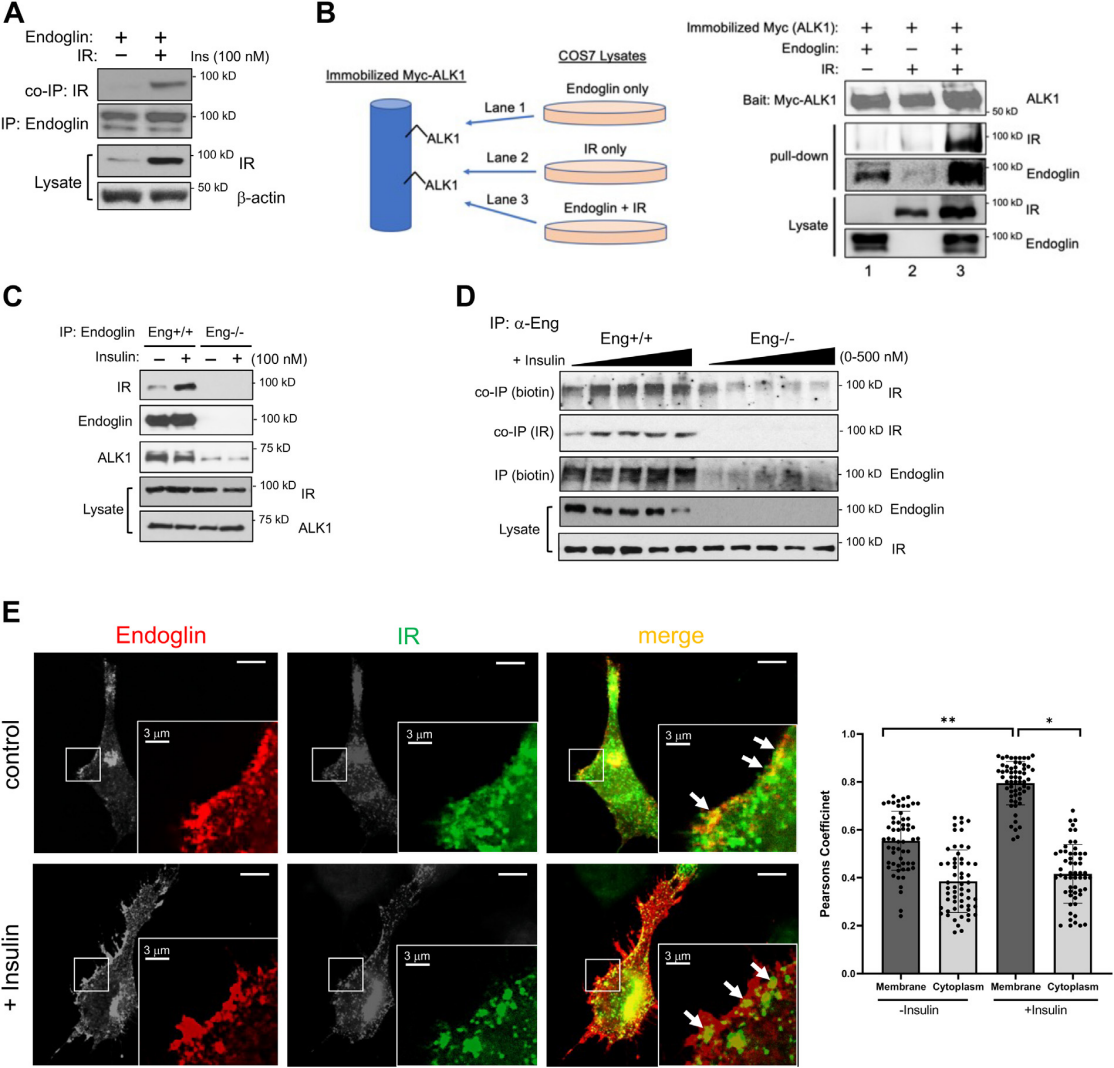
**Endoglin associates with insulin receptor (IR) at the cell surface.***A*, COS-7 cells overexpressing endoglin and IR were immunoprecipitated with endoglin antibody. Western blot shows IR co-IP (*top panel*) upon endoglin IP (*second panel*) ALK1 in the presence or absence of insulin stimulation for 15 min after 6 h of serum starvation. *B*, COS-7 cells transiently overexpressing Myc-tagged ALK1 alone was immobilized on anti-Myc bead column (schematic). An ALK1 pull down was performed using separate cell lysates prepared from COS-7 cells overexpressing endoglin alone, IR alone, or endoglin and IR. Western blots show the immunocomplex comprising ALK1 (*top panel*), IR (*second panel*), and endoglin (*third panel*). *C*, Western blot shows the co-IP of endogenous endoglin, IR, and Alk1 in the presence or absence of insulin stimulation for 15 min. Eng+/+ and Eng −/− ECs serum starved for 6 h were treated with insulin. Cell lysates were immunoprecipitated for endoglin (*second panel*) and observed for the co-IP of IR (*top panel*) and Alk1 (*third panel*). *D*, Eng+/+ and Eng−/− ECs serum starved for 6 h were treated with insulin (0, 50, 100, 200, and 500 nM) for 10 min prior to cell surface biotinylation. Cell lysates were immunoprecipitated for endoglin, then resolved on SDS-PAGE and blotted for biotinylated IR and endoglin (*top* and *third panels*, respectively) and total co-IP of IR (*second panel*). *Bottom two panels* show endogenous endoglin and IR in the cell lysates. *E*, representative immunofluorescence images of Eng−/− MEECs transiently expressing endoglin and GFP-tagged IR. Transfected cells were treated with or without insulin (30 min) before fixing and staining for endoglin (*red*). *Arrows* indicate the colocalization of endoglin and IR along the membrane (*yellow*). About 25 cells per group (three ROIs per cell) were quantified using Image J plugin JACoP to determine Pearsons correlation coefficient near the cell membrane and cytoplasm. The scale bar represents 10 μm. Error bars indicate mean with SD and type 2 *t* test result shows ∗*p* < 0.001; ∗∗*p* < 0.0001 relative to control (no treatment) or as indicated. The scale bar represents 10 μm. EC, endothelial cell; IP, immunoprecipitation; ROI, region of interest.

To do so, we initially overexpressed hemagglutinin (HA)-tagged endoglin and IR in COS7 cells prior to immunoprecipitation (IP) with an HA-antibody and observed their robust co-IP only when expressed together but not when IR was expressed alone (Fig. 2*A*). Because endoglin often exists in a complex with ALK1, the signaling receptor that phosphorylates Smad1/5, we next tested whether IR also interacts with ALK1. Here, Myc-tagged ALK1 was ectopically expressed in COS7 cells subsequently used as a bait by immobilizing it on an anti-Myc bead column. A pull down was then performed in which separately prepared cell lysates containing the ectopic expression of endoglin alone, IR alone, or endoglin and IR (Fig. 2*B*). Notably, results showed that ALK1 associated with IR only when endoglin was present, thus suggesting that endoglin acts as a crucial mediator in this novel ALK1–IR–endoglin complex. This trimeric interaction was further tested in an endogenous setting by using MEECs where endoglin IP resulted in the co-IP of endogenous ALK1, as well as basal interaction with endogenous IR (Fig. 2*C*). However, the basal endoglin/IR interaction was markedly enhanced when cells were briefly treated with insulin before the IP, whereas the endoglin–ALK1 interaction remained unchanged (Fig. 2*C*; third panel), suggesting that insulin augments the physical interaction between IR and the endoglin–ALK1 complex at the EC surface. To test this possibility, a cell surface biotinylation assay was performed on control and Eng−/− MEECs upon brief treatment with increasing concentrations of insulin prior to endoglin IP to assess for changes in cell surface endoglin/IR interactions (Fig. 2*D*). Here, we found that the biotinylated IR fractions coprecipitating with endoglin IP rose accordingly with increasing insulin concentrations, whereas no such detections were observed in Eng−/− ECs, thus supporting that insulin drives the cell surface endoglin-IR interaction in ECs (Fig. 2*D*).

Consistent with the aforementioned results, IF analysis revealed a distinct colocalization of endoglin and IR along the cell membrane when transiently coexpressed in Eng−/− ECs (Fig. 2*E*; *white arrows* and graph). As assessed by Pearsons correlation, insulin treatment only further enhanced their colocalization along the membrane but not in the cytoplasm, thus supporting a clear role of insulin in enhancing the endoglin/IR interaction at the cell membrane to activate Smad1/5 downstream.

### Insulin activates Smad1/5 through PI3K/Akt- and ERK-independent IR-Src signaling axis

**Figure 3:**
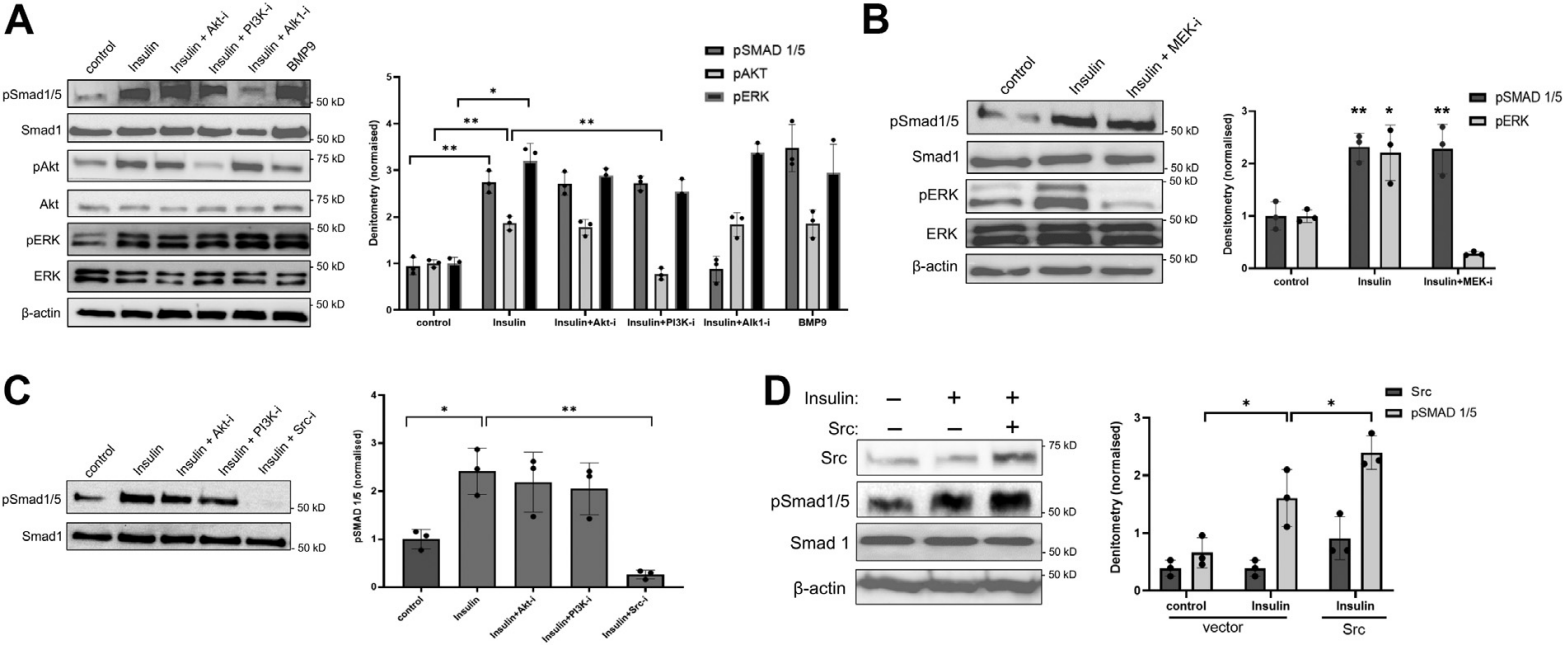
**Src promotes insulin-induced smad1/5 activation.***A*, Western blots show phosphorylated levels of Smad1/5, Akt, and ERK in Eng+/+ ECs treated with insulin (100 nM) alone or in the presence of 15 min pretreatment with small molecule inhibitors of Akt (Akt-i; 10 μM), PI3K (PI3K-i; 20 μM), or ALK1 (Alk1-i; 0.6 μM). BMP9 treatment was used as positive control (1 nM). Data are representative of three independent experiments. Graph shows densitometry analysis of the ratio of pSmad1/5 to total Smad1, pAkt to total, and pERK to total. Fold change of pSmad1/5, pAKT, and pERK levels relative to control (no treatment) cells are represented. Error bars indicate mean with SD and type 2 *t* test result shows ∗*p* < 0.008; ∗∗*p* < 0.001, compared to control or as indicated. *B*, Western blots show pSmad1/5 and pERK levels in Eng+/+ cells treated with insulin (100 nM) alone or in the presence of pretreatment with MEK inhibitor (MEK-i; 30 μM) for 15 min. Cell lysates were immunoblotted for pSmad1/5 and pERK. Blots of three independent experiments were analyzed by densitometry and the ratio of pSmad1/5 to total Smad1 and pERK to total ERK were quantified. Fold changes of pSmad1/5 and pERK expression relative to control (no treatment) cells are represented in the bar graphs. Error bars indicate mean with SD and type 2 *t* test result shows ∗*p* < 0.008; ∗∗*p*< 0.001 compared to control. *C*, Western blot shows pSmad1/5 in response to insulin (100 nM) alone or in combination with pretreatment with Akt-i, PI3K-i, or Src-i (15) min. Blots of three independent experiments were analyzed by densitometry and ratio of pSmad1/5 to total Smad1 was quantified. Fold change of pSmad1/5 expression relative to control (no treatment) cells is represented in the bar graphs. Error bars indicate mean with SD and type 2 *t* test result shows ∗*p* < 0.008; ∗∗*p*< 0.001 compared to control or as indicated. *D*, Eng+/+ ECs transfected with either vector control or Src (1 μg) was treated with insulin (100 nM) for 30 min. Cell lysates were immunoblotted for Src and pSmad1/5. Blots of three independent experiments were analyzed by densitometry and ratios of Src to β-actin and pSmad1/5 to total smad1 were quantified. Normalized values of Src and pSmad1/5 are shown. Error bars indicate mean with SD and type 2 *t* test result shows ∗*p* < 0.05 compared to control or as indicated. EC, endothelial cell.

Since insulin primarily activates the PI3K/Akt and ERK/MAPK pathways in ECs (12, 15, 18), we tested whether these prominent effectors are involved in Smad1/5 phosphorylation by using pharmacologic inhibitors of PI3K, Akt, and ERK. Importantly, while insulin proved capable of activating Smad1/5 as robustly as our positive control, BMP9, pretreatment of cells with a PI3K inhibitor had no effect on Smad1/5 phosphorylation (Fig. 3*A*; lanes 2 *versus* 4). Similarly, pretreatment of cells with a pan-Akt inhibitor, which blocks the Akt kinase activity irrespective of its phosphorylation level at S473, had negligible effect on insulin-dependent Smad1/5 induction (Fig. 3*A*; lane 3). But LDN193189, an inhibitor of the BMP pathway that demonstrates high selectivity for ALK1 at low concentrations (25), completely abrogated insulin-induced Smad1/5 phosphorylation, thus demonstrating that insulin requires IR, endoglin, and the ALK1 kinase to stimulate Smad1/5 phosphorylation (Fig. 3*A*; graph). To further show that insulin-induced Smad1/5 phosphorylation directly occurs through ALK1, we overexpressed a kinase-dead ALK1 point mutant (ALK1^K221R^), which exerted a dominant negative effect of abolishing Smad1/5 activation relative to control (Fig. S2*C*). Next, we tested how MEK inhibition, which blocks the downstream ERK activation, affects insulin-induced Smad1/5 phosphorylation (26). As is shown in Figure 3*B*, MEK inhibition failed to prevent Smad1/5 phosphorylation, whereas it effectively blocked ERK activation (Fig. 3*B*; panel 1 *versus* 3, and graph), thus suggesting that insulin-IR signals through a pathway other than the canonical PI3K/Akt and ERK pathways.

To determine whether the insulin-induced IR kinase activity and downstream IRS1/2 activation are involved in Smad1/5 phosphorylation, the IR tyrosine autophosphorylation and IRS1/2-activation were assessed in Eng+/+ and Eng−/− ECs (Fig. S3). Here, we noted that IR tyrosine phosphorylation levels were similar in both cell types in response to insulin stimulation (Fig. S3*A*), as was the activation of the downstream insulin receptor substrate (IRS) 1/2 upon insulin treatment, although surprisingly the basal tyrosine (Y612) phosphorylation level was greater in Eng−/− ECs (Fig. S2*B*). But notably, because the insulin-induced activation of IR/IRS1/2 in endoglin-null cells was nevertheless comparable to the WT ECs and yet incapable of causing Smad1/5 phosphorylation, these data suggested that IRS1/2 is not directly responsible for this effect. However, a previous study has shown that insulin stimulates the IR recruitment of the Src kinase, which in turn, promotes the tyrosine phosphorylation of IR as well as downstream signaling (27). Hence, we explored whether Src is involved in this process by using PP2, a broad-spectrum inhibitor of Src family kinases (27), and discovered that PP2 treatment completely blocked insulin-induced Smad1/5 activation compared to Akt and PI3K-specific inhibitors (Fig. 3*C*; graph). To confirm the role of Src, we ectopically expressed Src and found a direct correlation between Src expression and insulin-induced Smad1/5 phosphorylation (Fig. 3*D* and graph), indicating that the insulin-IR signaling axis requires Src activity to stimulate the endoglin/ALK1 complex for Smad1/5 phosphorylation.

Several studies have reported an important crosstalk between Src and TGF-β signaling, particularly in relation to the role of TβRII (28, 29, 30, 31). Because the endoglin–ALK1 complex requires TβRII to activate Smad1/5 in response to TGF-β but not BMP9, we examined whether TβRII is involved in Src-dependent Smad1/5 activation in response to insulin (Fig. S3*C*). Here, we observed that TβRII overexpression failed to enhance the insulin-induced Smad1/5 phosphorylation, thus suggesting that TβRII is not essential for this process.

### Quantitative proteomics analysis of insulin treated Eng+/+ and Eng−/− MEECs

**Figure 4:**
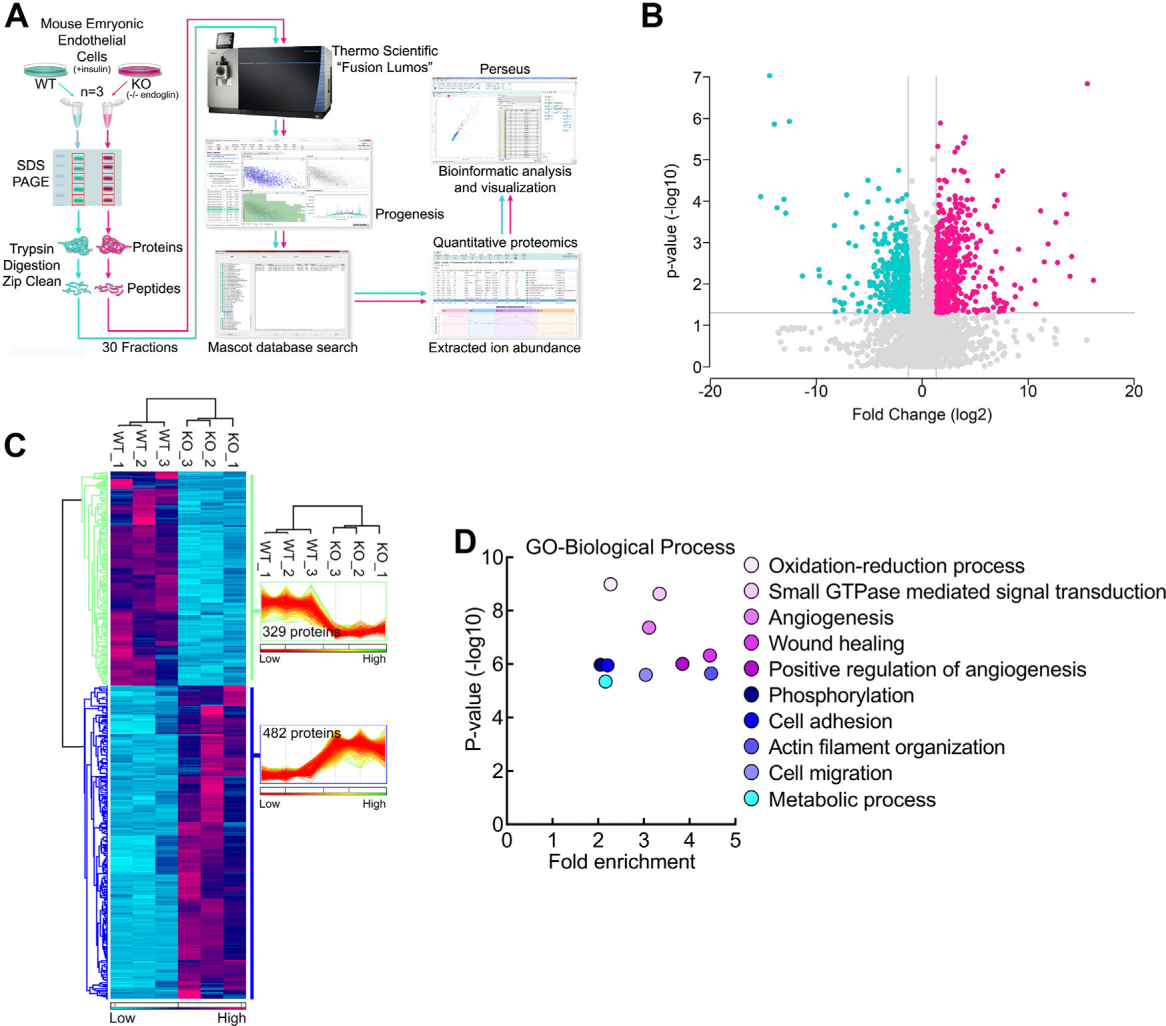
**Quantitative proteomics analysis.***A*, schematic shows the MS-proteomics workflow. Lysates collected from Eng+/+ and Eng−/− EC treated with insulin (100 nM for 16 h) (n = 3) were resolved by SDS-PAGE for gel-based fractionation followed by in-gel tryptic digestion, then subjected to tandem MS prior to data quantification using Progenesis QI for Proteomics and Mascot-based database searching. *B*, volcano plot of the proteins identified in the Eng+/+ and Eng−/− EC lysates. Above the *horizontal gray line* represents the cut-off for a *p* value of <0.05 while the two *vertical lines* represent the cut-off values of 2-fold change in either the positive or negative direction. *C*, unbiased hierarchical clustering of the 811 significantly affected proteins confirmed that the expression patterns across the different individual biological samples cluster together accordingly as either Eng+/+ and Eng−/−. A heat map and linked dendrogram of the hierarchical clustering results provide a visual representation of the clustered matrix and the associated profile plots further reveal consistency within groups of the corresponding protein expression patterns. *D*, scatter plots of the biological processes gene ontology enrichment findings for Eng+/+ and Eng−/− ECs upon insulin treatment. EC, endothelial cell; MS, mass spectrometry.

To identify proteins responsive to insulin-induced Smad1/5 activation associated with endoglin, we performed unbiased quantitative proteomics analysis on Eng+/+ and Eng−/− MEECs treated with insulin for 16 h (Figs. 4*A* and S4). Volcano plot analysis of all 7000-plus proteins identified in the quantitative proteomics experiment highlighted the finding that the loss of endoglin significantly alters insulin-induced protein expression changes within the MEECs, supporting the hypothesis that endoglin mediates transcriptional activation/repression within MEECs in response to insulin (Fig. 4*B*).

Unbiased hierarchical clustering and accompanying heat map visualization of a subset of the significantly affected proteins revealed that endoglin deletion results in both losses (329) and gains (482 proteins) in protein abundance relative to control (Fig. 4*C*), while gene ontology analysis of this group of significantly affected proteins (811) identified the highest enrichment in biological processes associated with angiogenesis, wound healing, actin filament organization, and cell migration (Fig. 4*D*), most of which can strongly influence cell migration, differentiation, and capillary morphogenesis during angiogenesis. Among the top statistically significant responders, ependymin-related 1 (EPDR1) was found to be highly upregulated in Eng+/+ cells with a 13-fold increase compared to Eng−/− MEECs (Fig. S4*B*).

### EPDR1 is a gene target of insulin-induced Smad1/5 activation

**Figure 5:**
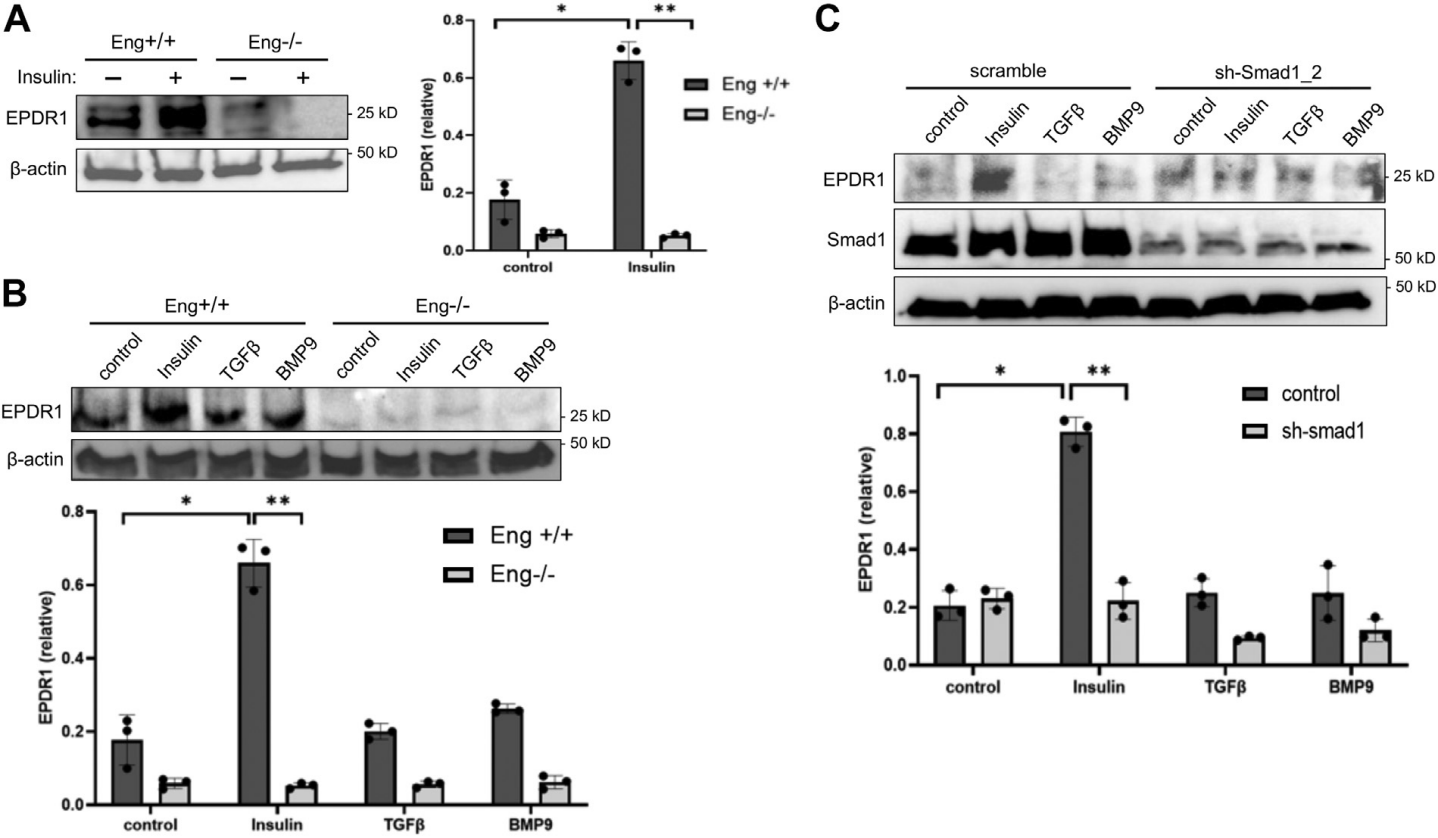
**EPDR1 is a Smad1 gene target induced by insulin but not TGF-β or BMP9.***A*, Western blot shows EPDR1 expression upon insulin treatment (100 nM) for 16 h in Eng+/+ and Eng−/− ECs. Blots of three independent experiments were analyzed by densitometry. Ratio of EPDR1 to β-actin was quantified and normalized EPDR1 level is demonstrated in the bar graph. Error bars indicate mean with SD and type 2 *t* test result shows ∗*p* < 0.001; ∗∗*p*< 0.0001 compared to Eng+/+ control or as indicated. *B*, Western blot shows EPDR1 expression upon treatment with insulin (100 nM), TGFβ (200 pM), or BMP9 (1 nM) treatment for 16 h in Eng+/+ and Eng−/− MEECs. Blots of three independent experiments were analyzed by densitometry. Ratio of EPDR1 to β-actin was quantified and normalized EPDR1 level is demonstrated in the bar graph. Error bars indicate mean with SD and type 2 *t* test result shows ∗*p* < 0.001; ∗∗*p*< 0.0001 compared to Eng+/+ control or as indicated. *C*, Western blot shows EPDR1 expression upon treatment with insulin (100 nM), TGFβ (200 pM), or BMP9 (1 nM) for 16 h in scramble control and sh-Smad1_2 ECs. Blots of three independent experiments were analyzed by densitometry. Ratio of EPDR1 to β-actin was quantified and normalized EPDR1 level is demonstrated in the bar graph. Error bars indicate mean with SD and type 2 *t* test result shows ∗*p* < 0.001; ∗∗*p*< 0.0001 compared to control (no treatment) or as indicated. EC, endothelial cell; MEEC, mouse embryonic EC.

EPDR1 is a cell adhesion molecule structurally related to protocadherins and ependymins (32, 33, 34, 35), although this protein has never been associated with insulin/IR or TGF-β/Smad signaling. Therefore, we first validated our quantitative proteomics results through immunoblotting where Eng+/+ cells showed more than 3-fold increase in basal and nearly 13-fold increase in insulin-stimulated expression of EPDR1 compared to Eng−/− MEECs (Fig. 5*A*; graph), thus confirming that this cell adhesion protein is a Smad1/5 gene target controlled by insulin. However, neither TGF-β- or BMP9-induced Smad1/5 activity was able to enhance EPDR1 expression relative to basal levels (Fig. 5*B*; graph), suggesting that EPDR1 is specifically an insulin-induced Smad1/5 gene target coordinated by the endoglin–ALK1–IR complex.

To test whether Smad1/5 are involved in insulin-induced EPDR1 expression, we generated stable Smad1 knockdown MEECs (sh-Smad1) (Fig. S5*A*) and assessed their ability to control EPDR1 expression (Figs. 5*C* and S5*B*). In response to insulin, scramble control ECs displayed a striking upregulation of EPDR1 expression relative to control or even TGF-β and BMP9, whereas the expression proved minimal in sh-Smad1 cells regardless of ligand treatment (Fig. 5*C*; graph). Together, these findings confirm that EPDR1 is a newly identified Smad1 gene target selectively regulated by insulin.

### Insulin-induced EPDR1 expression promotes EC migration and endothelial capillary tubule formation

**Figure 6:**
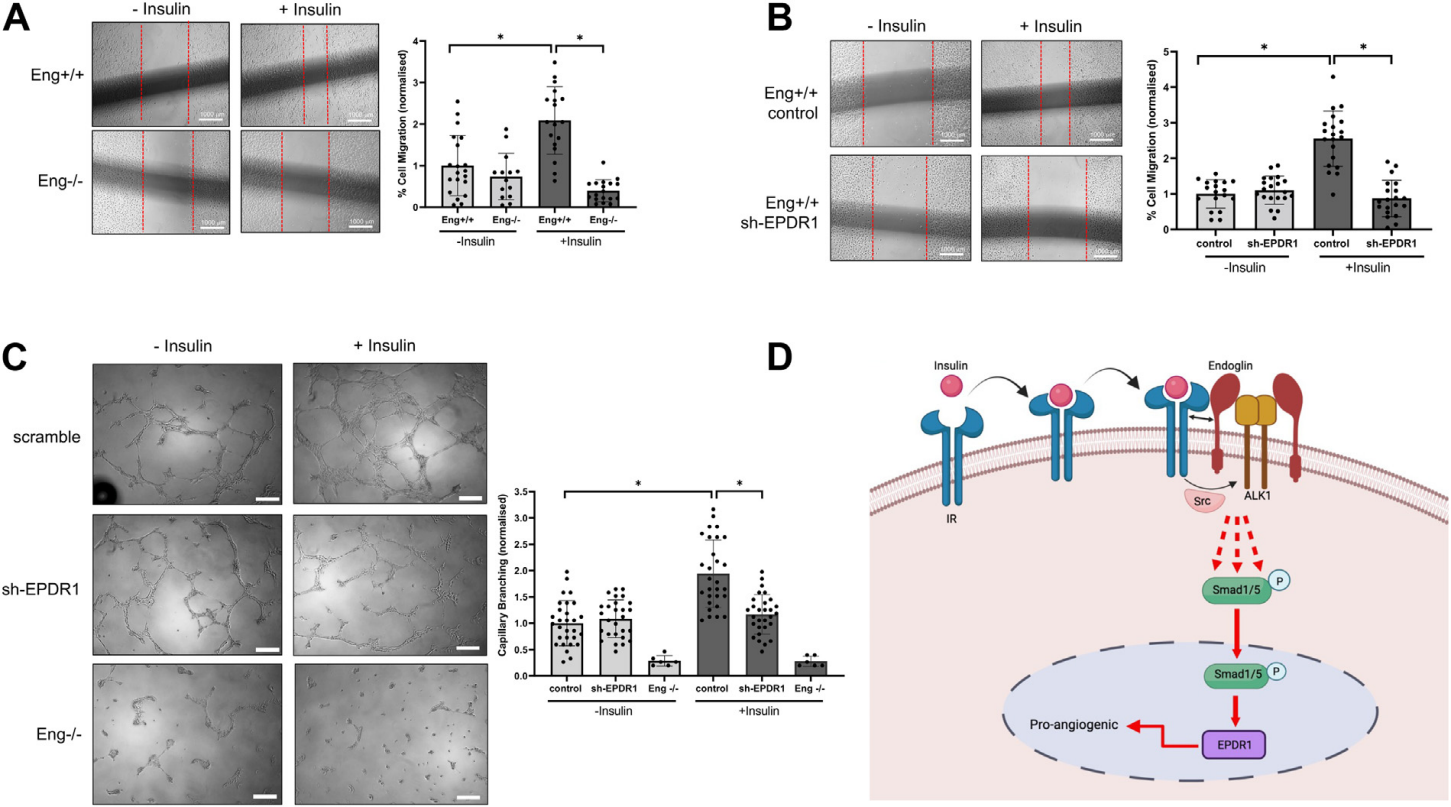
**Insulin-induced EPDR1 expression is required for migration and capillary-like branching in ECs.***A*, images show scratch-induced migration of Eng+/+ and Eng −/− ECs. Cells were allowed to migrate for 24 h in the presence or absence of insulin. Migration distance is measured in three different ROIs. Graph indicates percentage of migrated cells relative to Eng+/+ control cells based on three independent experiments. Error bars indicate mean with SD and type 2 *t* test result shows ∗*p*< 0.0001 compared to Eng+/+ control (no treatment) or as indicated. *B*, images show scratch-induced migration of scramble control and sh-EPDR1_2 ECs. Cells were allowed to migrate for 16 h in the presence or absence of insulin. Migration distance is measured in three different ROIs. Graph indicates percentage of migrated cells relative to scramble (no treatment) cells based on three independent experiments. Error bars indicate mean with SD and type 2 *t* test result shows ∗*p*< 0.00001 compared to scramble (no treatment) or as indicated. *C*, images show Matrigel-induced capillary-like branching in control, Eng−/−, and sh-EPDR1_2 MEECs at 16 h in the presence or absence of insulin. Graph indicates fold change in number of branches normalized to scramble (no treatment) from triplicates for each of the three independent experiments. The scale bar represents 200 μm. Error bars indicate mean with SD and type 2 *t* test result shows ∗*p*< 0.00001 compared to scramble (no treatment) or as indicated. *D*, a working model of how insulin activates Smad1/5 through the endoglin/ALK1/IR trimeric complex involving Src. This schematic was created with BioRender.com. EC, endothelial cell; MEEC, mouse embryonic EC; ROI, region of interest.

EPDR1 function remains poorly characterized, although it has been recently shown to modulate cell migration and invasion of cancer cells (32, 35). Given its new link to insulin and Smad1/5 signaling, we began our investigation by stably knocking down EPDR1 in Eng+/+ ECs (Fig. S6*A*). IF analysis showed that EPDR1 was localized primarily as perinuclear clusters that became more pronounced upon insulin stimulation, whereas stable knockdown cells displayed minimal signal (Fig. S6*B*; *red*). Next, we explored the role of EPDR1 in EC migration and angiogenesis by performing scratch wound healing and Boyden transwell assays to measure collective and single cell migration, respectively. To first determine how insulin affects EC motility in our system, we compared Eng+/+ *versus* Eng−/− MEECs under serum-deprived conditions in the presence or absence of insulin (Fig. 6*A*). Here, Eng+/+ cells displayed strong responsiveness to insulin as evidenced by the increased migration relative to control, whereas Eng−/− cells generally showed lower migration independent of insulin treatment (Fig. 6*A*, graph). In parallel experiments using EPDR1 knockdown Eng+/+ ECs, the migratory responses were all comparable to control at basal conditions but were sharply reduced (∼2.5-fold) in response to insulin (Figs. 6*B* and S6*C*; graphs). Similar patterns on cell motility were observed for these cells using the Boyden chamber studies (Fig. S7), thus supporting the notion that endoglin-dependent EPDR1 upregulation in response to insulin promotes EC migration.

Further consistent with the aforementioned conclusion, EPDR1-depleted ECs showed similar potential to form capillary-like tubules in Matrigel at basal conditions unlike Eng−/− ECs but notably failed to promote capillary branching in response to insulin when compared to control ECs (Fig. 6*C*; graph). Together, these results indicate that EPDR1 is a previously unknown effector of insulin signaling critical for angiogenesis and mediated by IR crosstalk with the endoglin–ALK1 receptor system in vascular ECs.

## Discussion

Our present work identifies an important crosstalk between IR and an endothelial TGF-β receptor complex that begins at the cell surface and culminates with the expression of EPDR1 as one of the major Smad1/5 gene targets critical for angiogenesis (Fig. 6*D*). This new signaling axis fundamentally differs from a previously proposed mechanism in which insulin increases TGF-β responsiveness by enhancing the delivery of TβRII and ALK5 to the cell surface, a widely expressed TGF-β receptor complex that activates the Smad2/3 pathway (22).

Several important mechanistic distinctions are noted between the insulin-induced activation of Smad2/3 *versus* Smad1/5. First, Smad2/3 activation still requires autocrine TGF-β signaling (22), whereas in the endothelial system, insulin itself appears to be the major driver of Smad1/5 activation through oligomerization of IR and endoglin–ALK1 at the membrane. We conclude that TGF-β does not play a significant role in this new signaling axis since Smad1/5 is rapidly activated by insulin (∼5 min) upon serum-deprived conditions (4–6 h). Second, insulin-dependent Smad2/3 induction appears to be mediated by Akt, which in turn promotes AS160 activation (22), an endosomal membrane trafficking protein known to promote the translocation of many transmembrane proteins to the cell surface (36, 37, 38, 39, 40). Our data, in contrast, demonstrate that the PI3K/Akt pathway is dispensable for Smad1/5 activation, and instead, requires insulin-IR–dependent Src activation (Fig. 3).

It is unclear how Src is involved in Smad1/5 phosphorylation, although it is plausible that Src enhances the activation of the ALK1 kinase through phosphorylation. Our efforts to identify potential Src-dependent tyrosine phosphorylation sites on ALK1 proved unsuccessful by mass spectrometry (MS) due to insufficient peptide coverage (data not shown), but nevertheless, this does not exclude the possibility that ALK1 is directly involved since its kinase activity appears to be essential. Previous studies have shown that Src is critical for TβRII activation—a process involving Tyr284 phosphorylation that mediates TGF-β-dependent mammary tumor growth and metastasis (28, 29). However, TβRII phosphorylation by Src reportedly had no effect on Smad activity, an observation consistent with our data indicating that TβRII is not essential for insulin-induced Smad1/5 phosphorylation. Instead, Src may modulate the activity of other ALK1 effectors such as CK2β, which we previously showed to directly interact with and promote ALK1 signaling in a phosphorylation-independent manner (41). While beyond the scope of the present study, precisely how the insulin-IR signaling axis mediates ALK1 activation through Src remains an important question to address to fully understand the role of insulin-Smad1/5 signaling during angiogenesis.

It is noteworthy that insulin-induced Smad1/5 activation drives EPDR1 expression but not TGF-β or BMP9 since they are both strong activators of this transcriptional pathway in the vascular system. However, while TGF-β and BMP9 can both regulate the same gene targets through Smad1/5 in ECs, distinct transcriptional responses have also been reported previously. For instance, BMP9 is thought to be a much more selective activator of ID1 transcription than TGF-β (5). A similar ligand-specific effect may be in play here where Smad1/5 couples with other insulin-dependent cofactors to recognize insulin-responsive gene elements.

There are several disease implications from our findings, chief among which is that vascular disorders arising from hyperinsulinemia may be perceived as not only an insulin signaling problem but one that also involves the TGF-β pathways. Indeed, while research has shown that hyperinsulinemia mediates pathologic angiogenesis through the proliferative actions of the ERK MAPK pathway (42, 43), chronically elevated levels of insulin may also trigger the expression of EPDR1 and potentially many others through the endoglin/ALK1/Smad1/5 signaling axis. For instance, chondroitin sulfate synthase 2 (44, 45) and prostaglandin F2 receptor-associated protein (46, 47, 48) are two of the top hits ranked higher than EPDR1, whereas others, including osteopontin (49, 50) and platelet-derived growth factor receptor beta (51, 52), represent promising candidates ranked slightly lower based on several parameters including fold changes over negative control and unique peptide counts identified (Fig. S3*B*). Future studies could explore whether these protein factors can serve as unique vascular targets during pathologic angiogenesis such as in diabetic retinopathy (53, 54, 55, 56, 57).

In summary, we conclude that EPDR1 is a crucial mediator of insulin-induced angiogenesis. Its Smad1/5-induced expression is driven by a direct crosstalk between IR and endoglin–ALK1, a process that occurs in activated ECs of angiogenic vessels. These findings unveil important therapeutic implications for EPDR1 and the TGF-β pathways in pathologic angiogenesis during hyperinsulinemia and insulin resistance.

## Experimental procedures

### Cell culture, plasmid, and transfection

Endoglin-null (Eng−/−) and control (Eng+/+) ECs were derived from WT and endoglin KO mice at E9 as previously described (58). MEECs were grown in MCDB-131 supplemented with 10% fetal bovine serum (FBS) (Gibco), 2 mM l-glutamine, 1 mM sodium pyruvate (Invitrogen), 100 μg of heparin (Sigma), and 50 μg/ml endothelial cell growth supplement (Sigma). Cells were cultured in flasks coated with 0.05% gelatin. COS-7 cells were cultured in Dulbecco's modified Eagle's medium (Gibco) supplemented with 10% FBS (Gibco). Transfections were achieved by using Lipofectamine 2000 as described according to manufacturer's protocol (Invitrogen). The HA-tagged endoglin construct was reported previously (59). GFP-tagged human IR (IR-β) was acquired from Addgene (plasmid #22286).

### Reagents and antibodies

Matrigel matrix was obtained from Corning. Insulin, human recombinant, zinc solution was purchased from Thermo Fisher Scientific. TGF-β1 and BMP-9 were obtained from R&D Systems. Inhibitors of ALK1 (LDN193189), PI3K inhibitor (LY294002), Src family inhibitor (PP2), and MEK inhibitor (PD98059) were obtained from Sigma–Aldrich. Pan-Akt inhibitor (GSK690693) was purchased from Selleckchem. The following antibodies were all purchased from Cell Signaling: P-Smad1/5/9, Smad1, P-Akt serine 473, P-p42/44 MAPK, Src, and IR. β-actin was purchased from Sigma–Aldrich, anti-HA was from Roche, and Endoglin (P3D1), Endoglin (A-8), ALK1, and EPDR1 antibodies were purchased from Santa Cruz Biotechnology.

### Protein expression and knockdowns

The shRNA constructs for mouse Smad1 and EPDR1 were purchased from Sigma. The shRNA targeting sequences for mouse Smad1 were as follows: 5′- TCCTATTTCATCCGTGTCTTA-3′ and 5′-TGGTGCTCTATTGTGTACTAT-3′. The shRNA for mouse EPDR1 was lentiviral transduction particles and the shRNA targeting sequences were as follows: 5′-GCGTTTATACAGCCAAGGATT-3′ and 5′-CACCAAACAGTGTGCAAAGAT-3′. Smad1 knockdown was generated by transfecting each mouse shRNA constructs into Eng+/+ ECs using Lipofectamine 2000. EPDR1 knockdown (sh-EPDR1) was achieved by infecting EPDR1 shRNA lentiviral particles into Eng+/+ ECs using 5 μg/ml Polybrene in regular growth media. For both Smad1 and EPDR1, stable knockdown was achieved by selecting with puromycin (5–10 μg/ml). Individual puromycin-resistant colonies were picked and scaled up as individual clones upon biochemical validation of endogenous >80% Smad1 depletion. Level of knockdown was tested comparing its endogenous protein level with that of scrambled control cells. Scramble control cells were transfected with scrambled control shRNA plasmid and were also selected with puromycin (5–10 μg/ml).

### Cell surface biotinylation

Cells were treated with or without insulin at various concentrations (0, 50, 100, 200, and 500 nM) for 10 min prior to being washed with PBS, then treated with 0.5 mg/ml biotin-LC (Pierce) on ice for 15 min. Upon extensive washes with PBS, cells were lysed with lysis buffer (20 mm Hepes, pH 7.4, 150 mM NaCl, 0.5% NP-40, 2 mM EDTA, 10 mM NaF, and 10% w/v glycerol) supplemented with protease and phosphatase inhibitor cocktails (Sigma), lysates were normalized for total protein content *via* Bradford, and then were immunoprecipitated with A8 antibody. All biotinylated proteins were resolved by SDS-PAGE and probed with streptavidin-horseradish peroxidase, A8, and IR antibodies.

### IP

Cells were washed with PBS, lysed on ice with lysis buffer for 20 min (20 mM Hepes pH 7.4, 150 mM NaCl, 5 mM NaF, 1% NP-40), and supplemented with protease and phosphatase inhibitors (Sigma). The lysates were centrifuged at 13,000 RPM for 10 min, and the supernatants were incubated with appropriate antibodies and agarose G or protein A agarose for 6 h at 4 °C. The IPs were then pelleted, washed three times with lysis buffer, and were stored in 2× sample buffer prior to Western blot analyses.

### Western blotting

Western blotting cell lysates were separated by SDS-PAGE and electrophoretic transferred onto the polyvinylidene difluoride membranes (Bio-Rad). Transferred membranes were blocked with 5% milk in Tris-buffered saline with Tween-20 (TBS-T) and then incubated with primary antibodies at 4 °C overnight. Following day, membranes were washed 3× in TBS-T buffer and incubated with the secondary antibody for 45 min at room temperature (RT). Membranes were washed 5× in TBS-T each for 5 min, then imaged by ChemiDoc Imaging system (Bio-Rad).

### IF microscopy

Cells grown on coverslip that were washed with PBS, fixed with 4% paraformaldehyde, permeabilized in 0.1% Triton X-100/PBS for 5 min, and then blocked with 5% bovine serum albumin in PBS containing 0.05% Triton X-100 for 1 h. Cells were incubated with appropriate primary and fluorophore-conjugated secondary antibodies for 1 h at RT, washed, and then mounted with ProLong Antifade (Sigma).

### Transwell migration assay

Eng+/+, Eng−/−, or sh-EPDR1 MEECs were serum starved for 5 h and then plated in serum-free MCDB131 media containing 2 mM L-glutamine in the presence or absence of insulin. Cells were seeded in the upper chamber of the transwells (Costar Corning Inc, 8 μM polycarbonate membrane 6.5 mm insert, 24-well plate), which were coated with 0.02% gelatin prior to plating. The bottom wells were filled with MCDB131 media containing 10% FBS. Cells were allowed to migrate for 16 h prior to cell fixation and staining of the nuclei of the migrated cells on the bottom side of the membrane. The transwell membrane containing the stained migrated cells were digitally imaged in three random fields per well and counted using ImageJ software (https://imagej.nih.gov/ij/) to tabulate cells as they were manually identified.

### Wound scratch assay

Eng+/+, Eng−/−, or sh-EPDR1 MEECs were seeded in 6-well plates and grown to confluency in complete growth medium prior to scratch induction using a 200 μl pipette tip. The cells were washed 2× with PBS, then treated with or without insulin in serum-free media containing 2 mM L-glutamine for 16 h. Images of the open wound were taken immediately after adding the insulin treatment in the open wound and again after 16 h to photograph the wound closure by the migrated cells over time. For quantification, three measurements of each monolayer sample were taken in three independent wounds per sample.

### Endothelial tube formation

96-well plates were coated with Matrigel (Corning) and incubated at 37 °C for 30 min. Following Matrigel polymerization, Eng+/+, Eng−/−, or sh-EPDR1 MEECs were seeded in serum-free media containing 2 mM L-glutamine in the presence or absence of insulin and were grown for 16 h. For each well, three microscope fields were selected randomly and were photographed digitally. Capillary branches were counted in each field. Experimental condition was normalized as percentage compared to WT cells with no treatment.

### In-gel tryptic digestions, MS, database searching, and quantitative protoemics

In-gel tryptic digestion and HPLC-electrospray ionization-MS/MS was performed exactly as previously described (60, 61). Tandem mass spectra were extracted from Xcalibur ‘RAW’ files and charge states were assigned using the ProteoWizard 3.0 msConvert script using the default parameters. The fragment mass spectra were searched against the *Mus musculus* SwissProt_2018_01 database (16,965 entries) using Mascot (Matrix Science; version 2.6.0) using the default probability cut-off score. The following search variables were used: 10 ppm mass tolerance for precursor ion masses and 0.5 Da for-product ion masses; digestion with trypsin; a maximum of two missed tryptic cleavages; variable modifications of oxidation of methionine and phosphorylation of serine, threonine, and tyrosine. Crosscorrelation of Mascot search results with X! Tandem was accomplished with Scaffold (version Scaffold_4.8.7; Proteome Software). Probability assessment of peptide assignments and protein identifications were made using Scaffold. Only peptides with ≥95% probability were considered. Progenesis QI for proteomics software (version 2.4, Nonlinear Dynamics Ltd) was used to perform ion intensity–based label-free quantification as previously described (62).

## Data availability

Raw MS data will be available upon request.

## Supporting information

This article contains supporting information.

## Conflict of interest

The authors declare that they have no conflicts of interest with the contents of this article.
