# Supplementary material for: EPDR1 is a noncanonical effector of insulin-mediated angiogenesis regulated by an endothelial-specific TGF-β receptor complex
Source: J Biol Chem. 2022 Jul 21;298(9):102297. doi: 10.1016/j.jbc.2022.102297 (PMC9396412; doi:10.1016/j.jbc.2022.102297)
Supplement: Supplemental Figures S1–S7 [file mmc1.docx]

Supplemental Figure 1


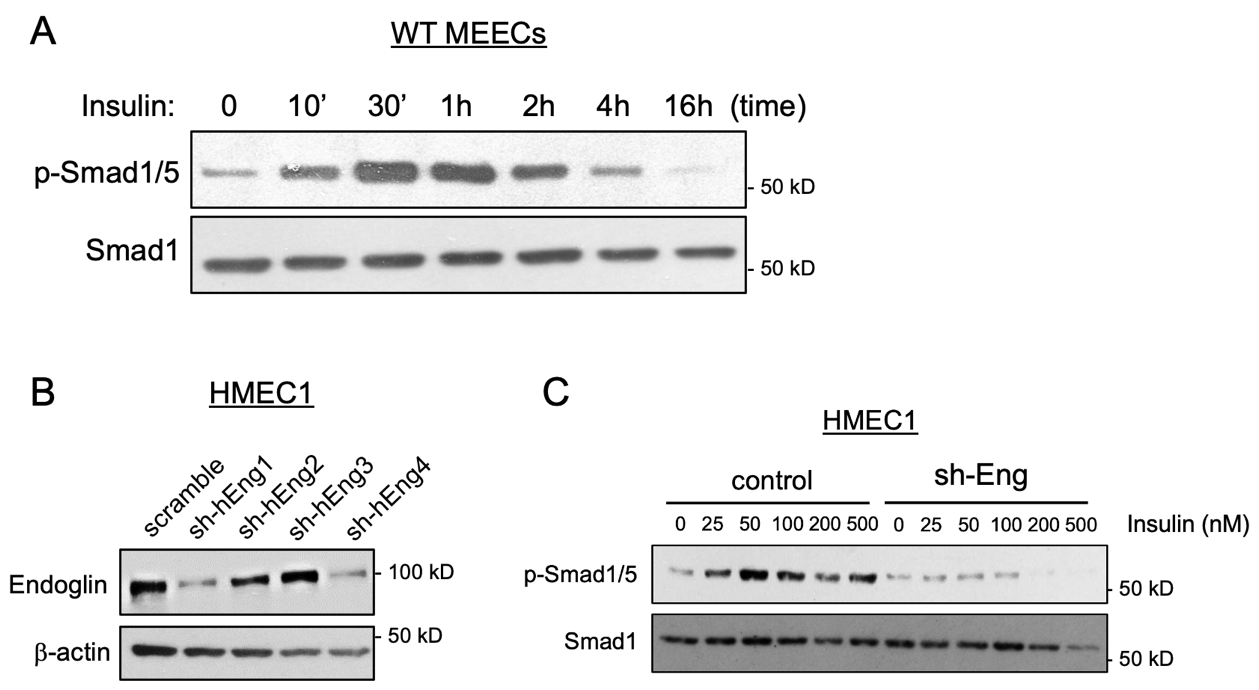


**S1**. **Insulin-induced Smad1/5 phosphorylation in MEECs and HMEC1s.**

A) Western blot shows p-Smad1/5 levels at indicated time points upon insulin (100nM) treatment in WT MEECs with prior 6 h serum starvation.

B) Western blot shows endogenous endoglin expression and stable knockdown using four distinct shRNA targeting sequences in HMEC1s. β-actin was used as loading control.

C) Western blot shows p-Smad1/5 levels in response to the indicated insulin concentrations for 30 min in control and sh-Eng4 HMEC1s.


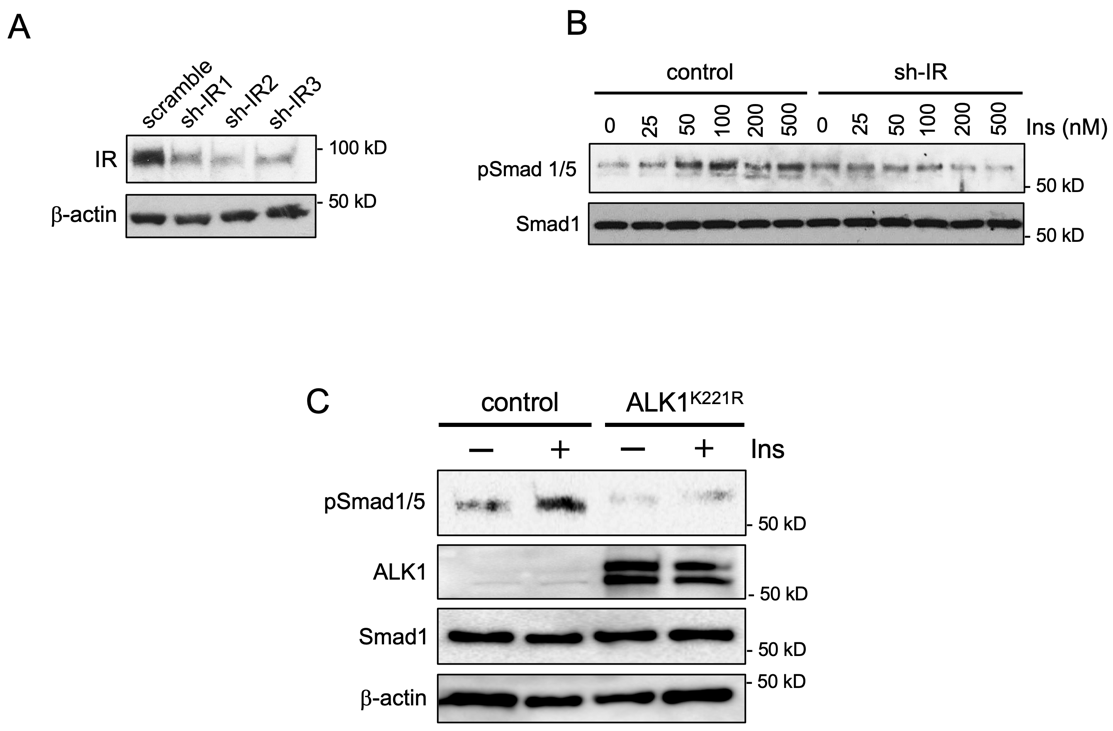
Supplemental Figure 2

**S2. Insulin-induced Smad1/5 activation requires insulin receptor (IR).**

A) Western shows the β-subunit of endogenous IR expression and its stable knockdown using three distinct shRNA targeting sequences in MEECs. β-actin was used as loading control.

B) Western blot shows pSmad1/5 levels in response to treatment with increasing insulin concentrations (30 min) in control and sh-IR2 MEECs.

C) Eng+/+ ECs transfected with either vector control or ALK1^K221R^ (2µg) were treated with insulin (100nM) for 30 minutes. Western blot shows p-Smad1/5, ALK1, total Smad1 and β-actin levels.

Supplemental Figure 3


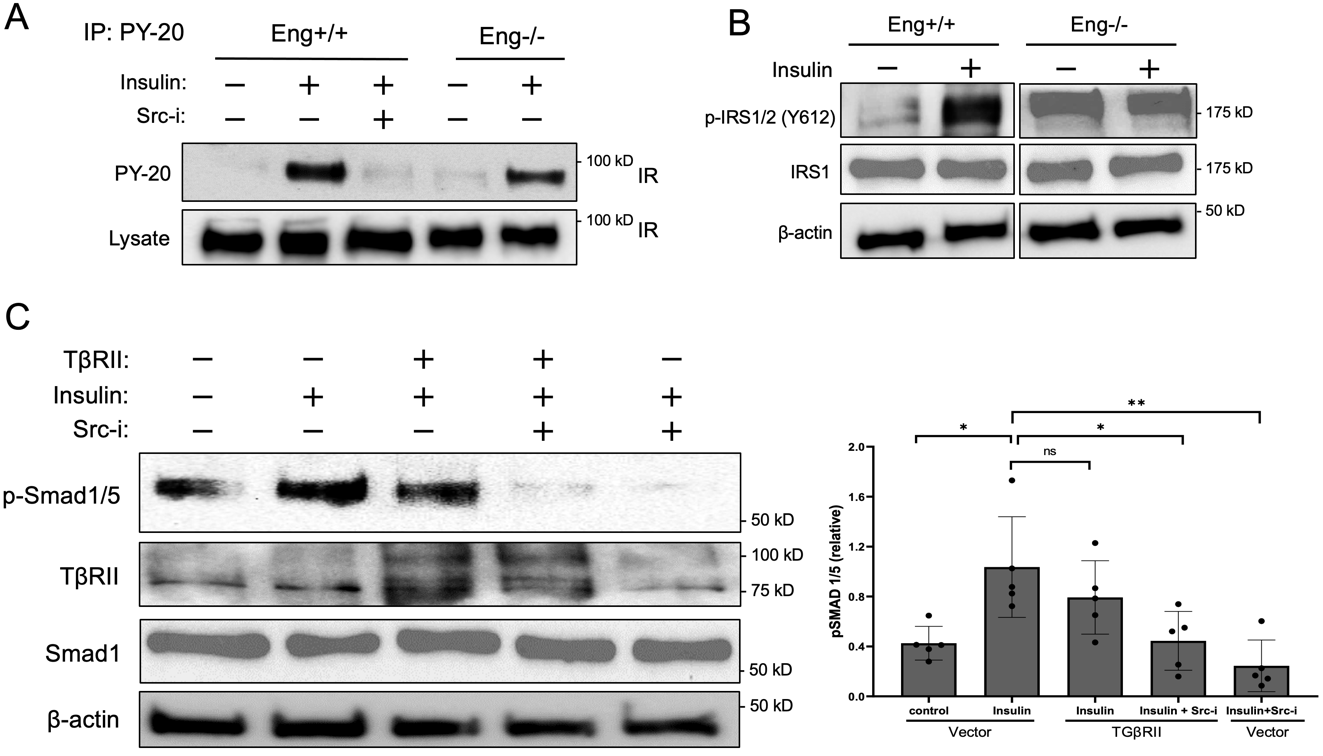


**S3. Determination of the role of insulin-induced IR kinase activity, downstream IRS1/2 activation, and TβRII in Smad1/5 activation in response to insulin.**

A) Eng+/+ and Eng-/- MEECs were treated with insulin (100nM) in the presence and absence of Src-i for 30 min. Western blot shows phosphorylated IR upon IPed for phosphorylated tyrosine-20 (pY-20) (top panel). Cell lysate shows total IR level (bottom panel).

B) Eng+/+ and Eng-/- MEECs were treated with insulin (100nM) for 30 min. Western blot shows phosphorylated IRS1/2 level when was immunoblotted for phosphorylated IRS1/2 at tyrosine 612 (p-IRS1/2 Y612) (top panel). Total IRS 1 and β-actin was used as loading controls.

C) Eng+/+ ECs transfected with either vector control or TβRII (2µg) were treated with insulin (100nM) in the presence and absence of Src-i for 30 minutes. Western blot shows p-Smad1/5, TβRII, total Smad1 and β-actin levels. Blots of three independent experiments were analyzed by densitometry and ratios of p-Smad1/5 to total Smad1 were quantified. Normalized values of p-Smad1/5 are shown in the graph. Error bars indicate mean with SD and type 2 t-test result shows *P<0.05, **P<0.005 compared to control or as indicated. *NS, non-significant*.

Supplemental Figure 4


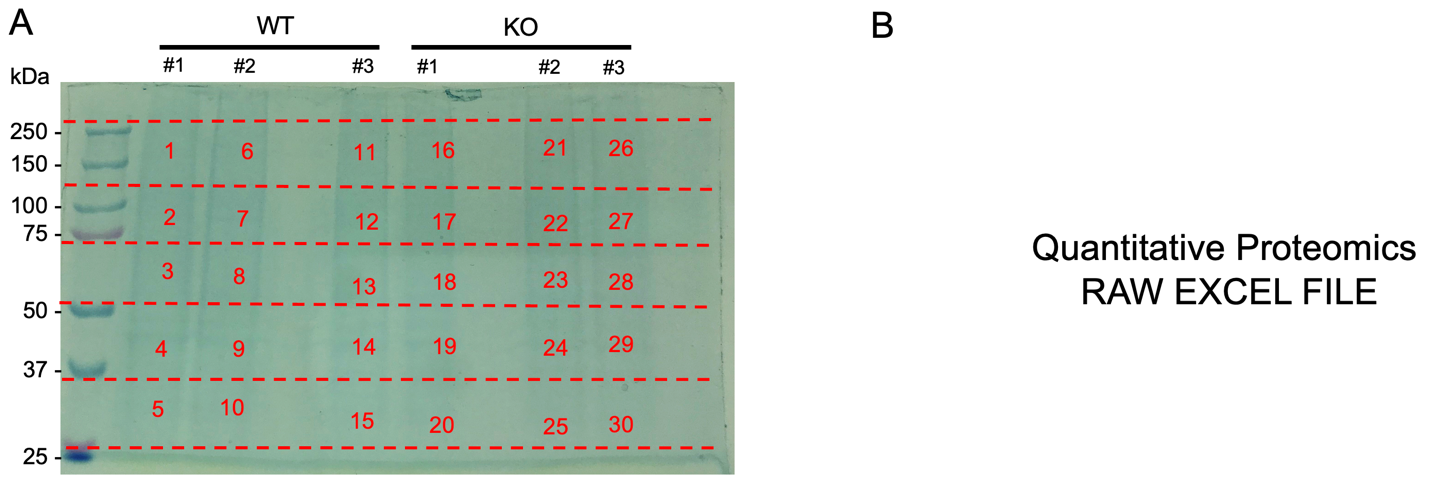


**S4. MS proteomics analysis in Eng+/+ and Eng-/- cells.**

A) Shown are the actual gel slices (n=3 per group) used for MS-proteomics from Eng+/+ and Eng-/- cell lysates resolved on SDS-PAGE.

B) Quantitative proteomics raw excel data file are shown separately.

Supplemental Figure 5


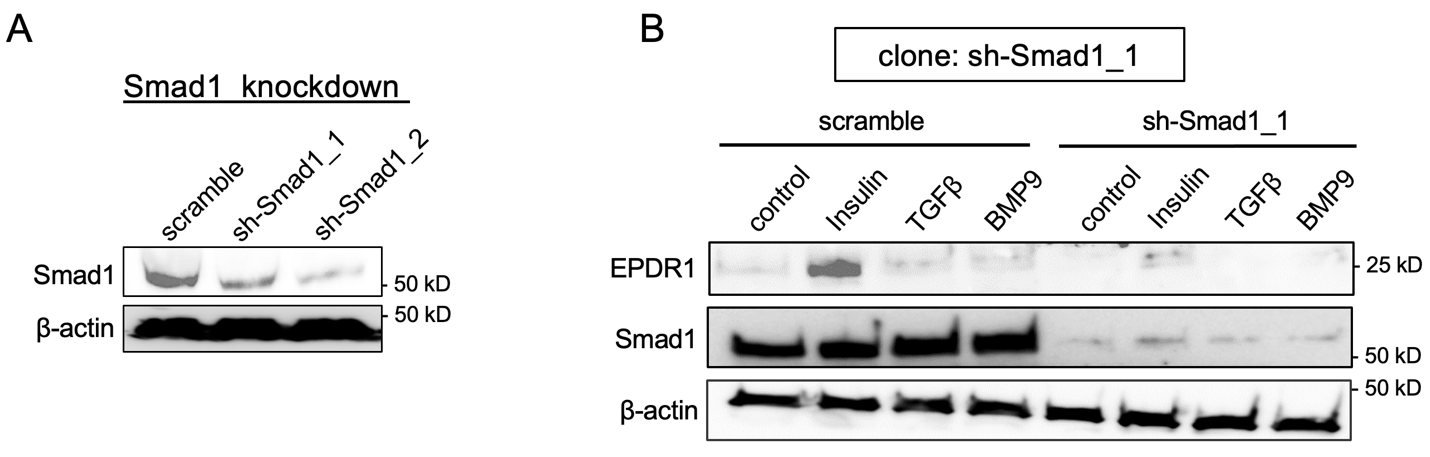


**S5. EPDR1 expression in Smad1 stable knockdowns in MEECs.**

1. Western blots show the endogenous expression of Smad1 and its knockdown using two distinct shRNA target sequences in MEECs. β-actin was used as loading control.
2. Western blot shows EPDR1 expression upon treatment with insulin (100nM), TGFβ (200pM) or BMP9 (1nM) for 16 h in scramble control and sh-Smad1_1 ECs. Smad1 level shows endogenous Smad1 expression and its knockdown. β-actin was used as loading control.

Supplemental Figure 6


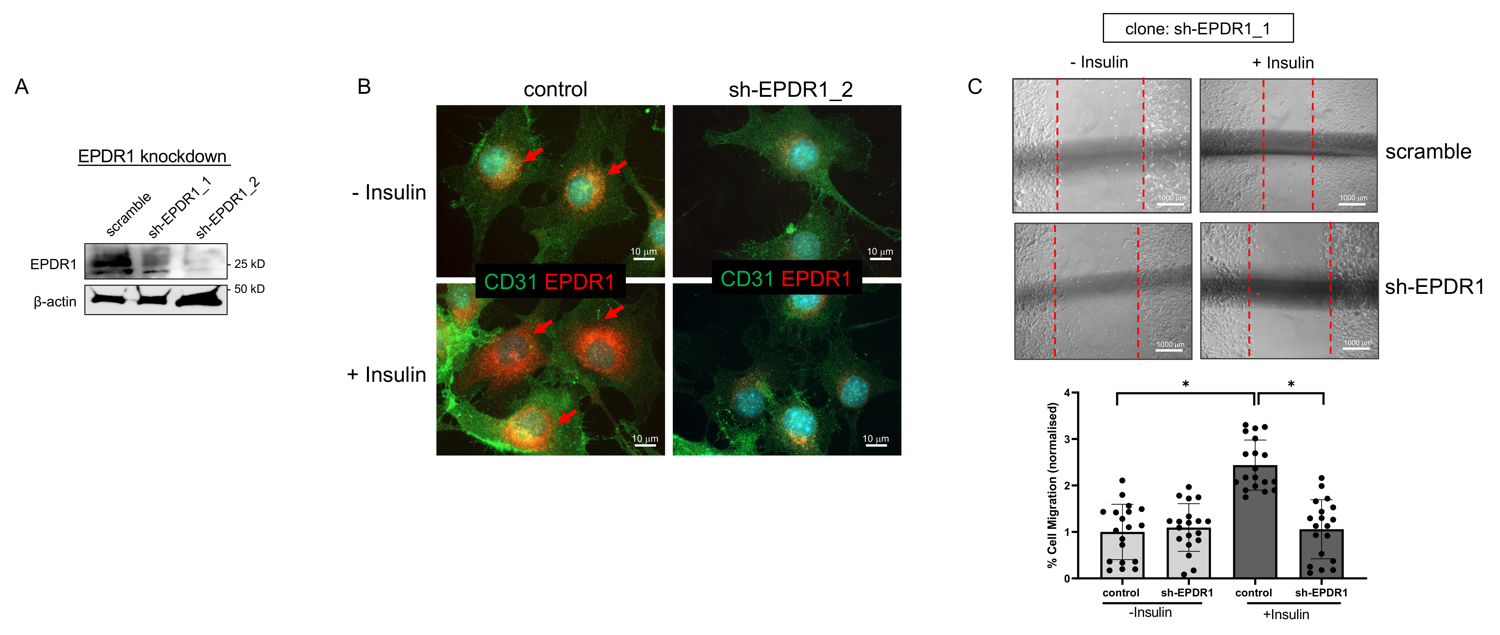


**S6. EPDR1 stable knockdowns in MEECs.**

1. Western blot shows endogenous EPDR1 expression and its knockdown using two distinct shRNA target sequences in MEECs. β-actin was used as loading control.
2. Representative immunofluorescence images show endogenous EPDR1 (red) and CD31 (green) in scramble control and sh-EPDR1_2 cells upon insulin treatment for 16 hr. Red arrows indicate EPDR1 distribution in the perinuclear region.
3. Representative images show scratch-induced migration of scramble control and sh-EPDR1_1 ECs. Cells were allowed to migrate for 16 h in the presence or absence of insulin. Migration distance is measured in three different ROIs. Graph indicates percentage of migrated cells relative to scramble (no treatment) cells based on three independent experiments. Error bars indicate mean with SD and type 2 t-test result shows *P< 0.00001 compared to scramble (no treatment) or as indicated.

Supplemental Figure 7


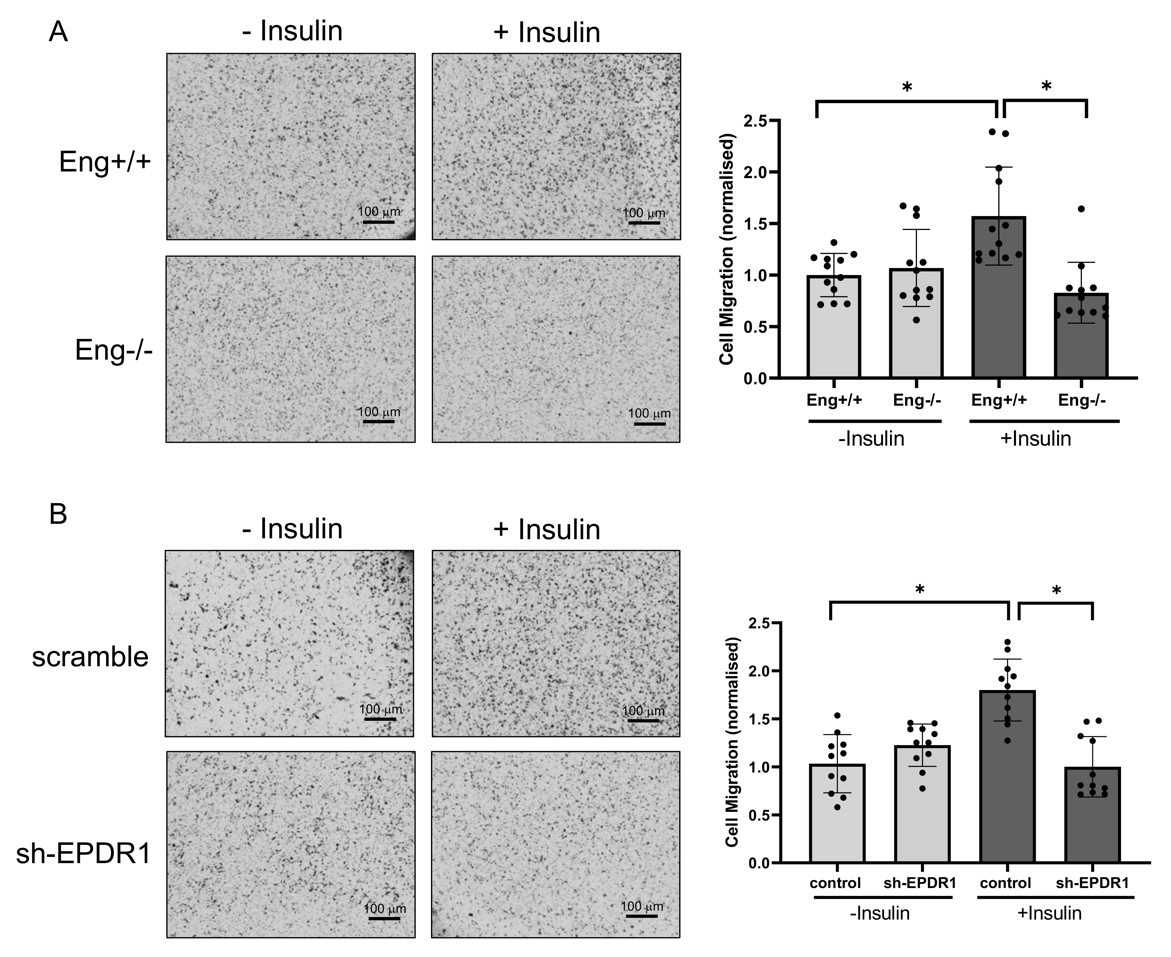


**S7. Insulin-induced EPDR1 expression is required for efficient migration.**

A) Images show transwell migration of Endo+/+ and endo-/- ECs in the presence or absence of insulin for 16 h. Number of migrated cells on the membrane bottom (shown as stained dots) were counted using ImageJ. Cell migration is represented graphically as a fold-change in number of migrated cells normalized to Eng+/+ control, from triplicates for each of the three independent experiments. Error bars indicate mean with SD and type 2 t-test result shows *P< 0.0001 compared to Eng+/+ control (no treatment) or as indicated.

B) Images show transwell migration of scramble control and sh-EPDR1_2 ECs in the presence or absence of insulin for 16 h. Number of migrated cells on the membrane bottom (shown as stained dots) were counted using ImageJ. Cell migration is represented graphically as a fold-change in number of migrated cells normalized to scramble (no treatment), from triplicates for each of the three independent experiments. Error bars indicate mean with SD and type 2 t-test result shows *P< 0.00001 compared to scramble (no treatment), or as indicated.
